# Knowledge, attitude and practices towards oral health among secondary school students in Huye district, Rwanda

**DOI:** 10.4314/ahs.v24i1.30

**Published:** 2024-03

**Authors:** John M Bayingana, Geofrey Shyaka, Japheths Ogendi

**Affiliations:** 1 Public Health, Rwanda polytechnic, Huye; 2 Public Health, Mount Kenya University, Kigali-Kicukiro-Rwanda, Thika, Central Kenya

**Keywords:** Knowledge, attitudes, practice, oral health

## Abstract

**Background:**

Good oral health knowledge is considered to be crucial for health-related practices and better oral hygiene.

**Objective:**

This study was aimed to assess knowledge, attitudes and practices towards oral health among secondary student in Huye district.

**Methods:**

A cross-sectional survey was conducted with 386 students from public secondary schools with advanced level. Boarding secondary schools were excluded. Stratified Clustering sampling technique was used for selecting study participants. A closed ended questionnaire was used for collecting data. Results were found on the basis of percentage and frequency using SPSS 21.0 version.

**Results:**

The majority of participants, 222 (57.5%) were female and 164 (42.5%) were male. The majority of the participants, 168 (43.5%) were between 15-17 years old. Out of the total population of students 1.8% had good knowledge, 56.2% had average knowledge and 42% had poor knowledge about oral health. Moreover, 56.2% had negative and 43.8% had positive oral health attitudes and overall practice towards oral hygiene of students showed that 74.6 % had poor practice and 24.4% had good practices.

**Conclusion:**

Oral hygiene has to be long life practice and oral health education have to be included as part of the school environment.

## Introduction

Oral cavity is the mirror of health in general as said Sir William Osler. There is evidence which suggested that the oral health is significantly linked to the general quality of human life 1. Oral health is considered as an immanent package of quality of life, self-esteem and the overall general health in human life. Thus, poor oral health can also be considered as major issue due to its impact on general health and quality of life. According to the report from World Health Organization (WHO), oral diseases, in general, affected 60-90% of school-aged children and very large majority of adult in most industrialized countries 2. Different surveys revealed that knowledge, attitudes and practices on oral health among students were low in developing countries compared to developed countries.

Most of the oral diseases are due to poor oral hygiene which are the results of poor oral knowledge and behaviors 2. Most of the adolescents believed that oral health consultation and treatment cost high which is considered as barrier to dental visit. The majority of the adolescents are known to visit the dentist only when they have pain 3, 4. Most of the researchers concluded that oral health education and awareness has to play a great role in raising up the knowledge on oral health and have suggested that it should be included in school curriculum. Education is considered as key in the process of developing attitudes that encourage and preserve healthy life 5. A survey conducted in Brazil reported that greater knowledge towards health incites attitudes transformation and being motivated to healthy behaviors that can increase self-sufficiency to execute tasks, quickening the outcome of interventions and promote health. Interesting adolescents to be the subject hood in health decisions and considering their needs and desire represents positive interaction between them and health professionals and facilitate a constructive health education program 6. Survey carried out in Tanzania revealed that oral education was included in the school curriculum which made them to have required knowledge on sign, causes and prevention measure of dental diseases, the burden of cigarette smoking and the importance of regular dental visit. On the frequency of food consumption, Carneiro said that most of respondent had an acceptable level of practices concerning their oral health. 7

In 2018, WHO published a report in which it emphasized on point that education and literacy have a great influence on peoples' knowledge, skills, aptitudes and attitudes and hence behaviors, which are very important health determinants 8. A study conducted in Nyamagabe district with the purpose of disease prevention, highlighted the basic things required for oral health care for Rwandan children the promotion of health had to be introduced in different school in order to address oral health problem in a sustainable and affordable way. Schools must be where healthy lifestyles are thought and practiced. When health education is made available and personnel hygiene (e.g., hand washing) is observed at school, hygiene practices will become routine for the students. Researchers believe that knowledge and participation from teachers, parents, and community will assist in maintaining a steady improvements on both oral and general health among students and their family members.^9^

The prevalence of dental pain among them is high and 70.6% of them had never visited dentist and other oral healthcare providers for treatment. The main reason of not visiting dental professional is mainly due to the high cost of care^10^.

According to the WHO, oral diseases are among the major non-communicable diseases (NCDs) that affect millions of people worldwide with low economic status and in Africa, around 80% of the population are affected. It impacts negatively on health, well-being, lifestyle and economic status of the population. Oral diseases are also source of pain, disfiguration and death. If oral diseases are detected early, they are either treatable or preventable. And the modifiable risk factors for major NCDs are shared with oral diseases. Improving oral health must be accompanied by control and prevention measures for NCDs among the population 11, oral health promotion of adolescent must be thought at school as school education. The accumulation of plaque and calculus during a time among children and adolescents is due to poor oral hygiene worldwide 12. Study showed that the preeminent place for a successful health education campaign is at schools because a large number of children from different ages are met there and if customized correctly, has good result in future 13.

Concerning activity restriction at school, children with poor oral health suffer more compare to those who are not suffered 14. Hours lost for children at school was around 50 million, due to the oral health problem and automatically, it is affected school performance of children and their late life success 15. Even if a good oral health is given by a knowledge but omitting the development in their attitudes and habit and makes it practical become a custom. Healthy behavior & lifestyles developed at a young age are more sustainable 17.

Oral health status in Rwanda showed that since 2008, the most cause of morbidity in district hospital was tooth and gum diseases, and dental decal prevalence 38.1% in Kigali among school-aged children 6–16 years 10. A study done by Morgan et al reported that 43% of study participants had suffered gingivitis, 22.2% had suffered from early periodontitis, 8.3% had suffered from advanced periodontitis and 3.1% had suffered from juvenile periodontitis. The major oral health problem in Rwanda are dental caries and gum diseases 10. Poor oral hygiene with increasing accumulation of plaque and calculus in the course of time has been reported among children and adolescents in both developed and developing countries 4

Nearly 4% of all outpatient consultations in Rwanda's community health centers and hospitals nationwide were related to patient chief complaints regarding oral infections, the mouth, or a tooth problem 23. Adolescent are receptive to a new information, giving them an adequate knowledge toward oral health should allow then to improve their oral hygiene practice and being disseminate in their family and their society 13. A study done at Wat Cantt revealed that only 5% and 9% of respondents had good knowledge and good practice on oral health respectively, 46% and 40% of them with average knowledge and practice respectively and 49% and 51% had poor knowledge and practice toward oral health respectively 13. Current oral health status among secondary students in Huye district was not known we conducted a study with the objective of determining knowledge, attitude and practice towards oral health among secondary school students.

## Methods

### Ethical consideration

In order to conduct this research, approval was sought from the Mount Kenya University (MKU) School of health science. The permission for conducted a survey in public school in Huye district were granted from Executive Secretary of the district with signed authorization letter. The purpose and objectives of the study was explained to the headmasters and received their agreement. The research objectives and methods were explained to the participants and verbal assent were obtained from students. They were assured that information collected was only for academic purposes and all respondents were told that their name is not needed on the questionnaires.

### Sample size

Given the nature of the study and the number of population study provided by ministry of education through Rwanda education statistics 2019.

Sample size was determined using Fischer Formula where


$$n=\frac{Z^{2}\;p(1-p)}{e^{2}}$$

$$n=\frac{\left(1.96{)}^{2}\;0.5\;(1-0.5\right)}{(0.05{)}^{2}}=385$$

Students were randomly enrolled in the study from all levels. The questionnaires were consisted of closed ended questions and the students were selected voluntary to complete it without sharing with their fellow students.

### Sampling technique

To obtain a representative sample, students from 12 sectors were selected in all district due to the selection of inclusive criteria. Stratified Cluster random sampling design was used to select 386 students from those public day school. Participants were grouped in different stratum as show below in the table then level of schools.

### Research design

The study was conducted in Huye district, 128 kilometers from Kigali city. Huye is located in southern province in Rwanda with the total surface area 581.6 square kilometers and density of 656.96 per square kilometer. Most of the population are farmers as their major activity 24.

A cross sectional study was conducted to assess oral health KAP through self-administered questionnaire.. Data from the study was collected in two months (May and June 2019) and analyzed.

### Target population

The target population was students from secondary schools who were from level one to level six, attending public secondary day schools in different sectors in Huye district. Public secondary schools are chosen because of their proximity and accessible by the local citizens of the sectors and their less bureaucratic procedure to be registered. Record from ministry of education in Huye district gave the estimation of 7463 students registered for 2019 academic years.

### Inclusion criteria

Only secondary school students from day schools with advanced levels.

### Exclusion criteria

All students from boarding secondary schools were excluded from the study because their food stuff is determined and restricted to their dietary behaviors.

### Data collection instrument

Data were collected using self-administered questionnaire in school settings and supervised by the school staff. The questionnaire was divided in four parts. First demographic characteristic of the participant, second knowledge towards oral health, third oral health attitudes and last practices towards oral health. A structured questionnaires was adopted from similar studies 1, 16, 4 modified and adopted in Rwandan context. A pilot study was done in order to check the validity of the tool. Questionnaires were translated in Kinyarwanda for easy reading and understanding.

### Procedure of data collection

The research instrument was structured and developed to gather KAP information towards oral health of study participants.

Headmasters were approached and presented letters form the district authorizing researcher to conduct a survey and the purpose of it was explained. Headmaster in collaboration with head-teachers and some teachers were selected students randomly, explained them the purpose of the survey and distributed questionnaires to be filled. After 20 minutes, questionnaires were collected and brought to data collector.

### Data analysis

Categorical data were entered in Excel 2013 and exported the SPSS version 21.0 and encoded then verified omission errors. Data analysis was descriptive statistics, percentage and frequency.

The first section of the questionnaire has three questions; one socio-demographic. Section two (eleven questions) highlights knowledge of students on gingival bleeding, dental appearance, flossing and number of teeth (deciduous and permanent). Each correct answer was score one and zero for wrong one but the eleventh question had multiple answers. All the scores were summed up. The responses to these questions were divided and categorized: poor knowledge (<7 marks), average knowledge (7-9) and good knowledge (>9). The third section (four questions) talked about students' attitudes toward oral health which covered the student's perception and dental care and their feelings and experiences during the dental visits. Positive attitude responses were scored one and negative attitudes and ‘I don't know response’ were scored zero. Participants who scored three good answers were considered to have a positive attitude and those scored less than three correct answers have been considered have negative oral health attitude. The section four, questionnaires contain twelve questions about oral health practices. Each correct answer was scored one and wrong answers were scored zero. All the scores were summed up. The responses to these questions were divided and categorized: poor oral hygiene practices (1-6 marks) and good oral hygiene practices (≥7 marks).

## Results

The general objective of the study was to evaluate the level of knowledge, attitudes, and practices towards oral health among students at secondary school in Huye district. Three specific objectives were deduced from the overall objectives including to evaluate the level of knowledge towards oral health among students of secondary school in Huye district, to assess the attitude towards oral health among students of secondary school in Huye district and to evaluate oral hygiene practice among secondary school students in Huye district.

### Oral health knowledge secondary school students

Objective one of the study was to determine the level of knowledge towards oral health among students of secondary school in Huye district.

**Figure 1:**
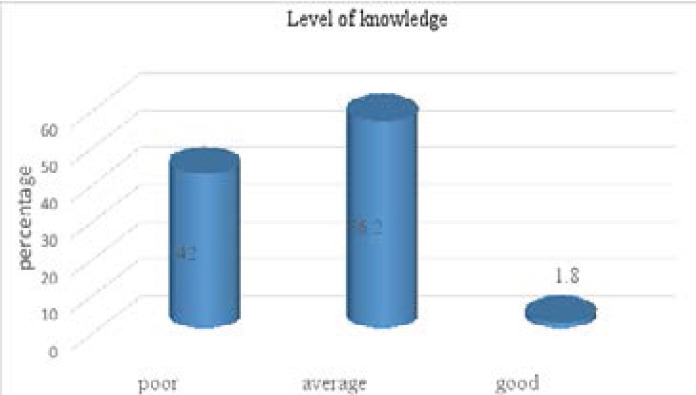
Level of knowledge of secondary school students

The level of oral health knowledge among students of secondary students were given in figure1. The finding revealed that 56.2 % of students had average knowledge and 42% had poor knowledge about oral health and only 1.8% had good knowledge.

### Oral health attitudes of school students

The results of oral health attitudes of students is presented in table 4. Individual and overall attitudes of respondent were estimated.

**Table 4:** Attitudes towards oral health of secondary school students

Variables	Frequency	Percentage
**To maintain healthy mouth is an individual responsibility**		
Yes	366	94.8
No	10	2.6
Don't know	10	2.6
**Periodic dental visit is important to maintain good oral health?**	333	86.3
Yes	26	6.7
No	27	7
Don't kwon		
**Dental treatment is it expensive?**		
Yes	93	24.1
No	195	50.5
Don't know	98	25.4
**Have you visited a dentist before?**		
Yes	218	56.5
No	168	43.5
**If yes, then for what reason?**		
Decay	14	3.6
Pain	101	26.2
Filling	21	5.4
Extraction	79	20.5
Any others specify (cleaning, orthodontic consultation)	3	0.8

**Source:** primary data.

**Figure 2:**
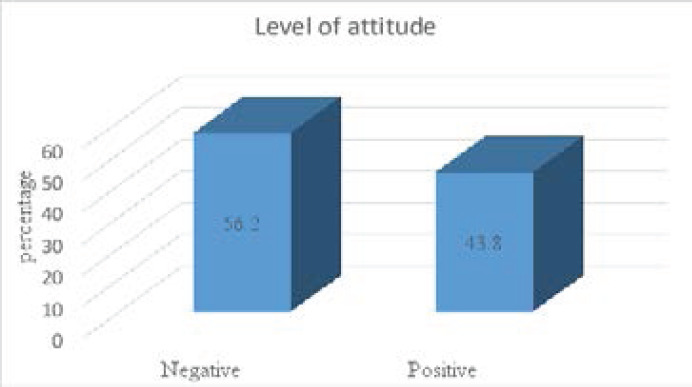
Level of attitudes of school students

The level of attitudes towards oral health was given in figure 2. The results showed that 56.2% of secondary students had negative attitudes and 43.8% of them had positive attitudes.

### Practices towards oral hygiene of school students

The results of oral health practices of students is presented in table 5.

**Table 5:** Practices towards oral health of school students

Variables	Frequency	Percentage
**Frequency of eating sweets?**		
Daily	33	8.5
3-4 times a week	41	10.6
Occasionally	289	74.9
Never	23	6
**How frequent do you take soft drinks?**		
Never/once in while	178	46.1
One a week	41	10.6
Twice a week	23	6
3 to 5 times/week	30	7.8
Everyday	114	29.5
**Times taken to brush your teeth?**		
Once daily	145	37.9
Twice daily	123	31.9
More than twice a day	35	9.1
After every meal	83	21.5
**The way you brush your teeth**		
Use horizontal strokes	112	29
Use vertical strokes	22	5.7
Both horizontal and vertical direction	159	41.2
Circular strokes	93	24.1
**How often do you change tooth brush?**		
One in 3 months	197	51
One in 6 months	41	10.6
One a year	21	5.4
When bristles get frayed up	108	28
Don't know	19	4.9
**The amount of paste you apply on your brush**	22	5.7
Full length of bristles	225	58.3
Half-length of bristles	139	36
Exactly Pea-sized		
**Do you press the paste in between the bristles?**		
Yes	272	70.5
No	114	29.5
**Do you rinse the mouth after taking meals?**		
Yes	323	83.7
No	10	2.6
Sometimes	53	13.7
**How do you clean your tongues?**		
Tongue cleaner	23	6
Fingers	18	4.7
Toothbrush	331	85.8
Any other specify	13	3.4
**How much time do you brush?**		
Less than a minute	42	10.9
one minute	108	28
two minutes	129	33.4
More than 2 minutes	107	27.7
**Besides the toothbrush and toothpaste, what do you use to keep your tooth clean?**	28	7.3
Chew stick twig	173	44.8
Charcoal	14	3.6
Tongue cleaner	158	40.9
None	13	3.4
Other specify		
**Frequency of dental visit**		
every 6 and 12 months	71	18.4
Sometimes	64	16.6
While dental pain	136	35.2
Never	115	29.8

**Source:** primary data.

**Figure 3:**
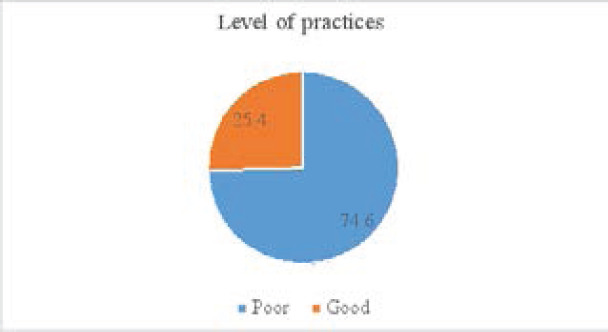
Level of practice towards oral health among students

Figure 3 revealed the level of practices among the participants. Results showed that 74.6 % of the respondents had poor practices and 25.4% of them had good practices towards oral hygiene.

## Discussion

The finding on level of knowledge towards oral health among students revealed that 42% of students had poor knowledge, 56.2% had average knowledge and only 1.8 % had good knowledge. In line with the present findings, study done in Visakhapatnam City 17 found that fair knowledge among secondary students was 97 % which was above compared to 56.2 % of this study and only 1% of them reported poor knowledge where this study revealed 42% of poor knowledge. In contrast, other study done in Tanzanian 7 revealed that 88.4% of the students had adequate knowledge towards oral diseases. The study done in Saudi Arabia 18, revealed that 66.5% of the participants had fair knowledge towards healthy oral hygiene. This result should be explained by the lack oral health or dental hygiene education program in schools. It can be due to misunderstanding about oral hygiene and the difficulty to access oral health services.

The finding of this study showed that more than half (56.2%) of participants had negative attitude and 43.8% of them had positive attitude. In line with the result from the present study, a study conducted in Karashi, Pakistan reported that about 50% of students had negative attitude towards oral health 19. In contrast with other studies, survey carried out in Kuching, Sarawak, reported 75.6% of participants had positive oral health attitudes and 24.4% of respondents had negative attitudes 5. Study done in Nigeria revealed that most of the respondents had positives attitudes on oral health status, as the most of them were strongly agreed or agreed on the importance of taking care of the mouth as other entire body by brushing twice a day 20. Positive attitudes can affect not only the oral hygiene behavior of students but also it can influence oral health behavior of the family and the community.

About 74.6% of students had poor practice on oral health. This is consistent with the study done in Ethiopia, reported that 61.6 % of participants had poor practice towards oral hygiene compared to this present study with 25.4 % of good practices 21. The poor oral health practice of this study is greater compare to the study carried out in Pakistan by 13 who reported that 51% of respondents had poor oral health practices, 34% and 40% of them had good and average practice towards oral health respectively. In contrast, the study done in Manipur, India, reported that 70.4% of respondents had adequate practice and 30% had poor oral practices towards oral health 22. The study strength is that participant was selected randomly and take in to consideration each sector in the district. The questionnaire was piloted to ensure the validity and translated in the mother tongue of the respondents. The choice of public day school was due to the fact that the majority if not all students come from low-income family. The study presents limitations such as limited sample size, only students were contacted and not parents which could be an occasion to have information on the health status on oral health from the family. Furthermore, the exclusion of students from the bonding school was the limitation of the study may be the result should be different.

## Conclusion

Secondary students in Huye district were a little knowledgeable towards oral health. The study findings showed that the level of knowledge of students was average (56.2%). The overall oral health attitudes shows that more than half of students had negative attitudes towards oral health. Overall result on oral hygiene practices among secondary students demonstrated that about three-quarter of them had poor practices towards oral hygiene.

## Figures and Tables

**Table 1:** Sample collected from students according to sectors

N^o^	Sectors	Number of students	Samples by sectors
**1**	Karama	828	(828*385)/7463 = 43
**2**	Tumba	1004	(1004*385)/7463 = 53
**3**	Kigoma	493	(493*385)/7463 = 26
**4**	Kinazi	818	(818*385)/7463 = 42
**5**	Maraba	722	(722*385)/7463 = 40
**6**	Mbazi	403	(403*385)/7463 = 21
**7**	Mukura	483	(483*385)/7463 = 46
**8**	Rusatira	457	(457*385)/7463 = 24
**9**	Rwaniro	494	(494*385)/7463 = 24
**10**	Simbi	413	(413*385)/7463 = 21
**11**	Gishanvu	445	(445*385)/7463 = 23
**12**	Huye	457	(457*385)/7463 = 24
	**Total**	**7463**	**387**

**Table 2:** Socio-demographic characteristics of secondary school's students

Variables	Frequency	Percentage
**Gender:**		
female	**222**	**57.5**
Male	**164**	**42.5**
**Age group (in years):**		
<12	9	2.3
12-14	76	19.7
15-17	168	43.5
18-20	107	27.7
>20	26	6.7
**Class:**		
S1	109	28.2
S2	77	19.9
S3	61	15.8
S4	59	15.3
S5	45	11.7
S6	35	9.1

**Source:** primary data.

**Table 3:** Oral health knowledge of secondary school students

Variables	Frequency	Percentage
**How many permanent teeth in adults' person?**		
20	5	1.3
28	8	2.1
32	372	96.4
26	1	0.3
**How many deciduous teeth are there?**		
20	92	23.8
28	63	16.3
32	220	57
26	9	2.3
**Occurrence of bleeding while brushing means?**		
Particles of food	32	8.3
Tartar/Calculus	92	23.8
Stains	36	9.3
Don't know	226	58.5
**A yellow or brownish color near tooth/gum means?**		
Gum infection	243	63
Healthy gums	8	2.1
Unhealthy gums	135	35
**To identify tooth decay/cavity**		
Hole and black spot in the tooth	220	57
Bleeding	29	7.5
Pain on tooth	115	29.8
Don't know	22	5.7
**Fluorides prevent tooth decay/cavity?**		
Agree	316	81.9
Disagree	34	8.8
Don't know	36	9.3
**Important of oral health compare to the overall health of our body?**		
Yes	367	95.1
No	19	4.9
**Reason of taking care of teeth and gums?**		
To smile good	16	4.1
Prevented to bad breath	98	25.4
To maintain teeth in healthy condition for long time	239	61.9
To reduce future cost of dental treatment…	32	8.3
**Can sweets and soft drinks affect teeth adversely?**		
Agree	283	73.3
Disagree	54	14
Don't know	49	12.7
**Can a dentist can polish and clean your teeth**		
Yes	333	86.3
No	52	13.5
The way to prevent dental problems? **		
Sticky foods and avoid sweets.	44	11.4
Brushing properly	138	35.8
Mouth rinsing after meals	3	0.8
Visit regularly a dentist	21	5.4
All of the above	180	46.6
** More than one answer		

**Source:** primary data.
